# Supplementary material for: Immune-Related Gene Profiles and Differential Expression in the Grey Garden Slug Deroceras reticulatum Infected with the Parasitic Nematode Phasmarhabditis hermaphrodita
Source: Insects. 2024 Apr 26;15(5):311. doi: 10.3390/insects15050311 (PMC11122010; doi:10.3390/insects15050311)
Supplement: Supplementary file 1 [file insects-15-00311-s001.zip › Table S1.pdf]

Table S1. List of primers used in this study for qRT-PCR.

Gene name	Forward (5'-3')	Reverse (5-3')
C-type lectin (CTL)	TGGACCCCAGTTTGTGTAAGAG	TACAAGTATATGGGTTGGCGGT
C-type lectin (CTL)/(CTLD)	TATATGCAGCGTCATCGTCAGA	AGCATCAACCCAACTCATCACT
Scavenger receptor (SR)	ACACTGGATAATGCTATTGCGC	GTCGTATTCAGGGAGGCAGATT
Perlucin-like protein	AGTCCAGTGATGACAGTGTGTT	GTGAGACAATGATGACAGAGCG
Akirin-2	ATCAGTTACAGAAGCGCTTTGG	CAATCTCCTTTCTCCTGAGGCA
Kunitz_textilinin-like	AGCCAACCTATTTTTGCGGC	GCGTGAAGACAGTTGTGGTG
Sialic acid-binding lectin 3	CCACAGGCCTTCTTAGCAATTT	CTCTGTGAACGTAGCAGAAACA
Immune-associated (IA)	TAAAGTTGAAGGGACCAGGACA	GACAGTTCCAAGTCCCTTCAGT
Dual oxidase (Duox)	TTCAAGGCTTTTTGGTTGACCC	AAACCAGGAAAACAGAGGAGCT
Toll-like receptor 4	TGGTACAACTTCTGCAACAGGA	ATGTTGTTCAAAGGGCGTGTTT
CD109 antigen	CTTCACTTTATTGCACTCCCCG	TGGAGTCACTGTCCAACAAGTT
C-type lectin (CTL)	AAGACTCAATTGGTTCGCCTCT	TGTTTTATTTCGCTCACCAGCC
Signal transducer	GAAAACTCCTGGCAAAGTCCAC	GTTTGCTGTAATTTGGGCGTTG
Immunoglobulin domain	AAATGAACTCCGCCTGTACAGT	TGTAGAATAGCGCCCTTTGTCA
Sialic acid-binding (Siglec-8)	CTCAATAGTTTCAGCGTATGGGC	GCCTCGTCCTGATAGTAACTGAA
Bactericidal/Permeability-IP	CGAATGTCAACATTGCTCTCAGT	CAAAATTTTGTAGCTTGCTCGCC
CD109 antigen-like gene	AGAGAAATCCTTCTGGTGGCTTT	ATTAAGGTTTGTGTCGGGTCTGA
Contactin-like Immunoglobulin	ACATTTCAGAGCACCGTCTCTAT	CAGTATCTGGCCCTCTGTTGTAA
Dret-Rpt6	TGCTGGAGCTCTTGAACCAG	TTCTTCCCGGACGCAAAAGA
